# Green preparation of reduced graphene oxide for sensing and energy storage applications

**DOI:** 10.1038/srep04684

**Published:** 2014-04-15

**Authors:** Zheng Bo, Xiaorui Shuai, Shun Mao, Huachao Yang, Jiajing Qian, Junhong Chen, Jianhua Yan, Kefa Cen

**Affiliations:** 1 State Key Laboratory of Clean Energy Utilization, Institute for Thermal Power Engineering, Department of Energy Engineering, Zhejiang University, Hangzhou, Zhejiang Province. 310027, China; 2 Department of Mechanical Engineering, University of Wisconsin-Milwaukee, 3200 North Cramer Street, Milwaukee, WI 53211, USA

## Abstract

Preparation of graphene from chemical reduction of graphene oxide (GO) is recognized as one of the most promising methods for large-scale and low-cost production of graphene-based materials. This study reports a new, green, and efficient reducing agent (caffeic acid/CA) for GO reduction. The CA-reduced GO (CA-rGO) shows a high C/O ratio (7.15) that is among the best rGOs prepared with green reducing reagents. Electronic gas sensors and supercapacitors have been fabricated with the CA-rGO and show good performance, which demonstrates the potential of CA-rGO for sensing and energy storage applications.

Graphene, a two-dimensional (2D) carbon material, has shown great promise in various applications due to its unique structure and properties^1^,^2^. To promote the practical applications of graphene-based materials, a priority should be given to the exploration for large-scale preparation of high-quality graphene with easy processing route and low cost. Up to now, diverse strategies have been applied for the production of graphene, mainly including mechanical or ultrasonic exfoliation^3^, chemical vapor deposition (CVD)/plasma-enhanced CVD (PECVD)^4^,^5^, epitaxial growth^6^, electric arc discharge^7^, chemical intercalation^8^, thermal/chemical reduction of graphene oxide (GO)^9^,^10^,^11^. Among these methods, chemical reduction of GO is recognized as a versatile and suitable method for the preparation of graphene in bulk quantities at a low cost. Unfortunately, a large number of widely used reducing agents are toxic and/or explosive, such as the commonly-used hydrazine hydrate (HH)^12^ and sodium borohydride^13^. As a consequence, continuous endeavors have been directed towards the development and optimization of eco-friendly reducing agents for GO reduction.

Recent studies revealed that some natural materials/chemicals are promising substitutes for toxic/explosive reducing agents for GO reduction, such as metals (*e.g.*, iron, zinc, and aluminum)^14^,^15^,^16^, alkaline solutions (*e.g.*, sodium hydroxide and potassium hydroxide)^17^, phenols (*e.g.*, gallic acid, Tannin acid, dopamine, and tea polyphenol)^18^,^19^,^20^,^21^, alcohols (*e.g.*, methyl alcohol, ethyl alcohol, and isopropyl alcohol)^22^, sugars (*e.g.*, glucose, fructose, sucrose, and natural cellulose)^23^,^24^, microbes (*e.g.*, *Escherichia coli* and baker's yeast)^25^,^26^, and other substances (*e.g.*, glycine, vitamin C, sodium citrate, and protein bovine serumalbumin)^27^,^28^,^29^,^30^. Generally, with eco-friendly reducing agents, the reduction of GO was successfully demonstrated to alleviate the environmental issues. However, challenges/problems still exist with the above green reduction processes. For example, inevitable impurities may remain in the products when using metals as the reductants^14^,^15^,^16^ and fairly low deoxygenation of GO can be obtained due to the poor reducing ability of gallic acid (C/O ratio of 3.89–5.28)^19^, tea polyphenol (C/O ratio of 3.1)^20^, and methanol (C/O ratio of 4.0)^22^. In addition, for some reductants, a rigid/harsh reduction condition was required, *e.g.*, alkaline environment is needed for sugars, dopamine, protein bovine serumalbumin^21^,^24^,^29^, and ionic liquids are needed for natural cellulose^23^. To this end, there is still a strong need to further explore novel green reducing agents for clean and effective reduction of GO.

Caffeic acid (CA, 3,4-dihydroxycinnamic acid, C_9_H_8_O_4_) is one of the most predominant hydroxycinnamic acids in the species of phenolic compounds, a group of substances recognized as excellent antioxidant. The chemical structure of CA can be described as two adjacent hydroxyl groups on an aromatic ring attached to the highly conjugated propenoic side chain. Previous studies have shown that the high antioxidant capacities of CA were attributed to the presence of hydroxyls at positions 3 and 4; and the two hydroxyl groups in CA contributed to a further increase in antioxidant potential by the donation of the hydrogen atom^31^. As a consequence, CA has been widely used as an effective antioxidant for applications ranging from the storage of soybean biodiesel^31^, the prevention of cardiovascular disease and cancer for human^32^, to the reduction of Cr(VI) for soil-plant system^33^. Since CA has excellent antioxidant activity and is widely available in plants and food^34^,^35^, it is reasonable to consider CA as a green, effective, and low-cost deoxygenation agent for GO reduction.

Inspired by the above facts, we herein, for the first time, propose a green and facile method for the chemical reduction of GO using CA as the reducing agent. The reduction of GO was successfully performed with a simple procedure, while the reduction level of the reduced GO (rGO) is among the best for green reducing reagents with a high C/O ratio of 7.15 (Supplementary Information, Table S1). The restoration of the electrical properties of graphene, *e.g.*, the high conductivity, through reduction is critical for applications of the rGO. Therefore, CA-reduced rGO (CA-rGO)-based electronic gas sensors and supercapacitors were demonstrated. The gas sensors with CA-rGO as the sensing material exhibited fast responses and high sensitivities to low-concentration NO_2_ (100 ppm) and NH_3_ (1%). For the supercapacitor application, electric double-layer capacitors (EDLCs) were fabricated using CA-rGO as the active materials. The capacitors showed substantially higher specific capacitances than those of GO due to the enhancement in active material conductivity, confirming the high reduction efficiency of CA. The demonstrated device applications confirm the successful preparation of rGO using CA as the reducing agent. Compared with previously reported reducing agents, CA shows advantages in terms of high reduction efficiency, low level of residual impurities, mild reaction conditions, and most importantly, environmentally friendly fabrication procedure. It is believed that this approach has great potentials for low cost and large-scale production of graphene-based materials from graphite.

## Results

**Figure 1:**
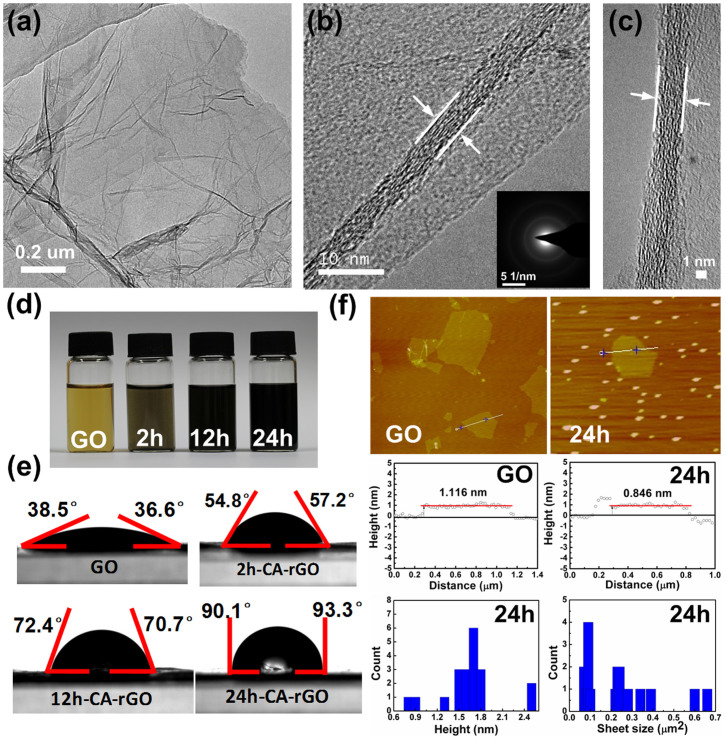
Structure characterizations of GO and CA-rGO. (a) TEM and (b, c) HRTEM images of 24h-CA-rGO. Inset: SAED patterns. (d) Digital photographs of aqueous dispersions of GO before and after reduction by CA for different reaction time. (e) Water droplet on the surface of GO and CA-rGO sheets. The error in the contact angle measurements is on the order of 0.1% of the measured values. (f) Tapping-mode AFM images and the corresponding height profiles of GO and 24h-CA-rGO dispersed on a mica substrate. About twenty 24h-CA-rGO sheets were characterized by AFM (Supplementary Information, Fig. S1). The height and size distributions of the 24h-CA-rGO sheets were obtained from the data shown in Fig. S1.

The prepared rGO samples were characterized to understand the rGO structure and the reduction efficiency of CA. Graphene and rGO are 2D nanosheets and usually bear transparent and wrinkled features under a microscope. Figs. 1a–c show the transmission electron microscopy (TEM) and high-resolution TEM (HRTEM) images of the rGO reduced with CA for 24 hours (24h-CA-rGO). After CA reduction, the intrinsic features of rGO such as large (few-micron size), transparent, and thin nanosheets with typical wrinkled and scrolled structure were observed. The HRTEM images show that the prepared rGO samples are few-layer (around 6–8 layers) nanosheets. Previous studies have shown that the thickness of the GO sheet may decrease after reduction due to the removal of the oxygen groups in the GO carbon plane^9^. To reveal the thickness change in the GO sheet, atomic force microscopy (AFM) imaging and thickness measurements on GO and rGO sheets were carried out. Fig. 1f shows the AFM images and height profiles of GO and 24h-CA-rGO. The thickness of GO measured from AFM data was 1.116 nm, in accordance with the thickness values of single-layer GOs reported in previous literature^20^,^23^. The thickness of the single-layer 24h-CA-rGO was around 0.846 nm, which is obviously smaller than that of the single-layer GO sheet, suggesting the effective reduction of GO with CA. To obtain reliable height and size information of the 24h-CA-rGO, AFM images were taken for around twenty 24h-CA-rGO sheets (Supplementary Information, Fig. S1). The height and size distributions of 24h-CA-rGO obtained from the AFM data are shown in Fig. 1f. Results indicate that the as-obtained 24h-CA-rGO is a mixture of single-layer and multilayer sheets and the size of the sheets is in the range of 0.07 to 0.66 μm^2^.

Fig. 1d shows the color change of GO suspension over the reaction time from 2 to 24 hours. The yellow brown GO suspension changed its color to black, indicating the reduction of GO^25^. Completely black homogeneous suspension was obtained for 24h-CA-rGO, suggesting the restoration of aromatic graphene structure^36^. As shown in Fig. 1e, GO exhibited highly hydrophilic nature as a result of sufficient oxidation. The contact angle of water on GO surface was 36.6°/38.5°, confirming the high wettability of GO in aqueous solutions. In contrast, the 2h-CA-rGO, 12h-CA-rGO, and 24h-CA-rGO samples showed increased hydrophobicity with a contact angle in the range of 54.8°–93.3°, which is in agreement with the previous work^37^. The increase in contact angle can be ascribed to the removal of oxygen functional groups in GO sheets, and the increased contact angle with different reaction times suggests that a higher reduction level of CA-rGO was achieved with a longer reaction duration.

**Figure 2:**
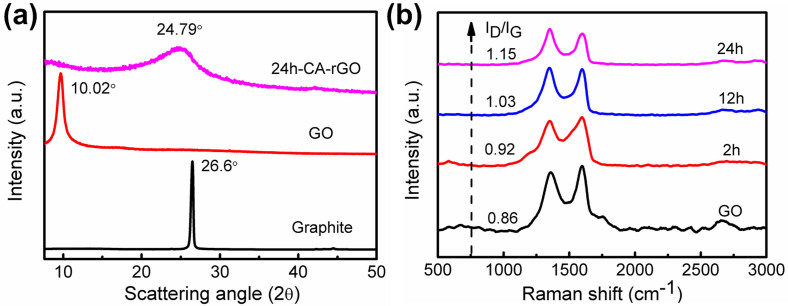
XRD and Raman data of GO and CA-rGO. (a) XRD patterns of pristine graphite, GO, and 24h-CA-rGO. (b) Raman spectra of GO before and after CA reduction for different reduction time.

To understand the atomic structures and interlayer spacings of the GO and rGO samples, X-ray diffraction (XRD) was carried out and the results are shown in Fig. 2a. The graphite exhibits a basal reflection (002) with a strong and sharp peak at 26.6° (corresponding to a d-spacing of 0.335 nm). Due to the oxidation of pristine graphite, the diffraction peak of GO shifts to a lower angle of 10.02° (corresponding to a d-spacing of 0.880 nm). As for the 24h-CA-rGO, the reflection peak at 10.02° disappeared while a broad peak centering at 24.79° (corresponding to a d-spacing of 0.359 nm) was observed. The relatively larger d-spacing of GO than that of pristine graphite is due to the intercalation of water molecules and the formation of oxygen-containing functional groups between the layers of graphite^5^. However, after reduction, the d-spacing of rGO was greatly decreased, indicating the removal of oxygen-containing functional groups^18^.

The structure configurations of GO and rGO samples were further investigated by Raman spectroscopy, as shown in Fig. 2b. Typically, two main bands exist in the spectra of graphite and graphene-based materials, *i.e.*, the G band assigned to the first-order scattering of the *E_2g_* phonon from sp^2^ carbon (graphite lattice), and the D band resulting from the structural imperfections created by the hydroxyl and epoxide groups on the carbon basal plane^38^. The G peak positions of three samples were in the order of graphite (~1,575 cm^−1^) < 24h-CA-rGO (1,586 cm^−1^) < 12h-CA-rGO (1,590 cm^−1^) < 2h-CA-rGO (1,596 cm^−1^) < GO (1,599 cm^−1^). The intensity ratio of D to G peak I_D_/I_G_ was in the order of GO (0.86) < 2h-CA-rGO (0.92) < 12h-CA-rGO (1.03) < 24h-CA-rGO (1.15). The increase of I_D_/I_G_ ratio after reduction is commonly found in GO chemical reduction studies^12^,^27^,^39^,^40^,^41^,^42^. It can be attributed to a decrease in the average size of the sp^2^ domains upon reduction of the GO, in which new graphitic domains were created that have smaller sizes than the ones present in GO before reduction, but are larger in quantities. Therefore, although there are more defect-free sp^2^ carbons after reduction, these carbons form smaller domains than those in the GO, which leads to large quantities of structural defects^12^,^42^. Another possible reason is the increased fraction of graphene edges, which could also contribute to the increase in the I_D_/I_G_ ratio^27^. To better understand the structure of CA-rGO, Fourier transform infrared (FTIR) and ultraviolet–visible (UV-vis) absorption spectra (Supplementary Information, Fig. S2) were included and the results further confirm that the GO was successfully reduced by CA.

**Figure 3:**
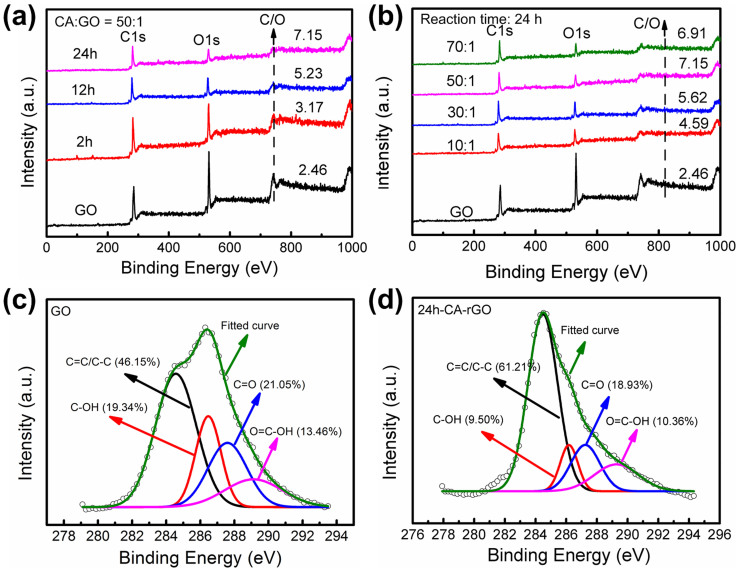
XPS data of GO and CA-rGO. (a) XPS survey spectra of the as-prepared GO and CA-rGOs (CA:GO = 50:1) for different reaction times. (b) XPS survey spectra of the as-prepared GO and CA-rGOs with different CA to GO ratios (reaction time: 24 hours). Gaussian line fitted C1s spectra of (c) GO and (d) 24h-CA-rGO.

The GO reduction level was also investigated by X-ray photoelectron spectroscopy (XPS) measurements. Fig. 3a shows the XPS survey spectra of GO and rGO samples (CA:GO = 50:1). As the reduction time prolonged, the C/O ratio increased from 2.46 (GO), to 3.17 (2h-CA-rGO), 5.23 (12h-CA-rGO), and 7.15 (24h-CA-rGO). The results indicate the removal of oxygen-containing groups in the CA-rGOs and the reduction level increased with the reaction time. The C/O ratio of 24h-CA-rGO sample was close to that using hydrazine monohydrate (C/O of 10.3)^12^, and much higher than those using tannin acid (C/O of 2.44)^18^, tea solution (C/O of 3.10)^20^, natural cellulose (C/O of 5.47)^23^, baker's yeast (C/O of 5.90)^25^, L-Ascorbic acid (C/O of 5.70)^43^, and gallic acid (C/O of 5.28)^19^. To investigate the impact of the CA:GO ratio on the reduction level, experiments with different ratios of CA to GO (10:1, 30:1, 50:1, and 70:1) for a fixed reduction time (24 hours) were carried out. The XPS results (Fig. 3b) show that the C/O ratio of CA-rGO increased with the increasing CA:GO ratio. For instance, the C/O ratio increased from 4.59 to 5.62 and 7.15 when the CA:GO ratio increased from 10:1 (pH = 5.4) to 30:1 (pH = 5.0) and 50:1 (pH = 4.7), respectively. However, the C/O ratio showed a very small change (from 7.15 to 6.91) when the CA:GO ratio increased to 70:1 (pH = 4.5). Therefore, a CA:GO ratio of 50:1 (pH = 4.7) and a reaction time of 24 hours are determined to be optimum for the CA reduction of GO. Figs. 3c and d show the Gaussian line fitted C1s spectra of GO and 24h-CA-rGO. In the spectra, four peaks centering at 284.6 eV (C = C/C–C), 286.5 eV (C–OH), 287.6 eV (C = O), and 289.1 eV (O = C–OH) are found, corresponding to different functional groups. The C–OH, C = O, and O = C–H peaks indicate the existence of oxygen-containing groups in the GO, *e.g.*, hydroxyl, epoxide, and carbonyl. After reduction (Fig. 3d), the intensities of C–OH, C = O, and O = C–H peaks greatly decreased, accompanied by an increase of the sp^2^ carbon peak, revealing that a large number of oxygen-containing groups were removed and the majority of the sp^2^ carbon networks were restored. Based on the above XPS results, the reduction of GO was confirmed by the significant decrease in the oxygen contents in GO. Thermogravimetric analysis (TGA) (Supplementary Information, Fig. S3) was also carried out to study the amount of oxygen groups in rGOs and the results show that the 24h-CA-rGO has a much smaller mass loss at elevated temperatures than GO, further proving that GO was reduced with CA. The reduction mechanism of CA was widely studied as a four-electron release process^33^, in which the release of four electrons can be expressed by the formation of semiquinonic radicals easily oxidizable to quinonic groups, whose further oxidation leads to the formation of carboxylic groups.

## Discussion

Most applications of graphene rely on its high electrical conductivity and unique structure. However, GO is non-conductive because of the extensive presence of saturated sp^3^ bonds, the high density of electronegative oxygen atoms bonded to carbon, and other “defects”^44^. Therefore, restoration of the high conductivity of graphene sheet is critical for its applications. In this study, two CA-rGO-based devices, *i.e.*, gas sensor and supercapacitor, have been developed and demonstrated for environment and energy applications. The results from the demonstrated device applications show that the rGO produced with CA has a high conductivity, and the reported method may serve as a simple and efficient method for green production of graphene.

**Figure 4:**
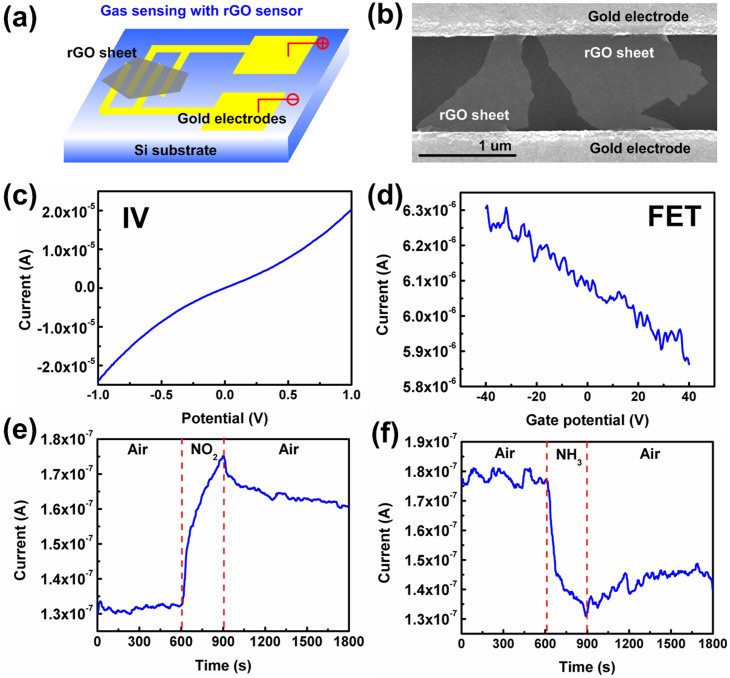
Gas sensor application of CA-rGO. (a) Schematic diagram of the rGO-based gas sensor device. (b) SEM image of 24h-CA-rGO sheets bridging a pair of gold sensor electrodes. (c) Direct current measurement results of 24h-CA-rGO with drain-source potential ramping from -1.0 to +1.0 V. (d) FET results (*V_d_* = 0.5 V) of the 24h-CA-rGO. Dynamic gas sensing results of the 24h-CA-rGO gas sensors for (e) 100 ppm NO_2_ and (f) 1% NH_3_ tested under room temperature.

Figs. 4a and b show a schematic of the rGO-based gas sensor and the scanning electron microscopy (SEM) image of the rGO sheets on the sensor electrodes. In this type of gas sensor, the rGO works as the sensing channel and the working principle of the gas sensor is based on the charge/electron transfer between the adsorbed gas molecules and the rGO sheet^45^. In general, by measuring the resistance/conductivity change of the rGO sheet in different gases, the presence and the concentration of the gas could be determined. Before the sensor was tested in different gases, the resistance of the 24h-CA-rGO device was measured with direct-current (dc) measurements, as shown in Fig. 4c. Based on dc measurement results, the 24h-CA-rGO sensor shows a resistance of 10^4^ to 10^5^ Ω, which is much smaller than that of GO (10^10^ Ω)^44^, with a linear I–V curve, indicating the reduction of GO. To study the transistor properties of rGO, field-effect transistor (FET) measurements were carried out in air. The gate potential (*V_g_*) dependence of the drain current (*I_d_*) of the 24h-CA-rGO sensor shows that the rGO was a p-type semiconductor (Fig. 4d), and the *I_d_* decreased when *V_g_* ramping from negative to positive. The FET results are in accordance with previous studies of rGOs^9^,^46^ and the on-off current ratio of the rGO sensor is relatively low compared with semiconducting carbon nanotubes and nanowires, which is because the rGO has a small bandgap and the 24h-CA-rGO has a multiple-layer structure.

Figs. 4e and f show the dynamic responses (*I_d_* vs. time) of the 24h-CA-rGO sensor exposed to 100 ppm NO_2_ and 1% NH_3_ diluted in air. The sensor was first exposed to a clean air flow for 10 minutes to record a base resistance; then a target gas flow was injected into the sensing chamber for 5 minutes to register a sensing signal; and finally a clean air flow was injected for 15 minutes to recover the sensor. From the sensing results, the sensor showed fast responses to both gases and the sensor resistance decreased with NO_2_ exposure and increased when exposed to NH_3_. The difference in the sensor response is because the NO_2_ and NH_3_ work as electron acceptor or donor in the sensing reaction and extracts electrons from rGO or injects electrons into rGO, respectively, thereby increasing or decreasing the rGO conductivity. The 24h-CA-rGO sensor has a sensitivity of ~1.33 (ratio of device resistance in air to that in target gas) to 100 ppm NO_2_ and ~1.35 (ratio of device resistance in target gas to that in air) to 1% NH_3_, which are similar to our previous reports^44^,^46^. The results from the sensor demonstration show that the CA-rGO can be readily used in sensor applications without any additional treatment and the performance of the sensor could be further improved through surface functionalization of the rGO sheet.

**Figure 5:**
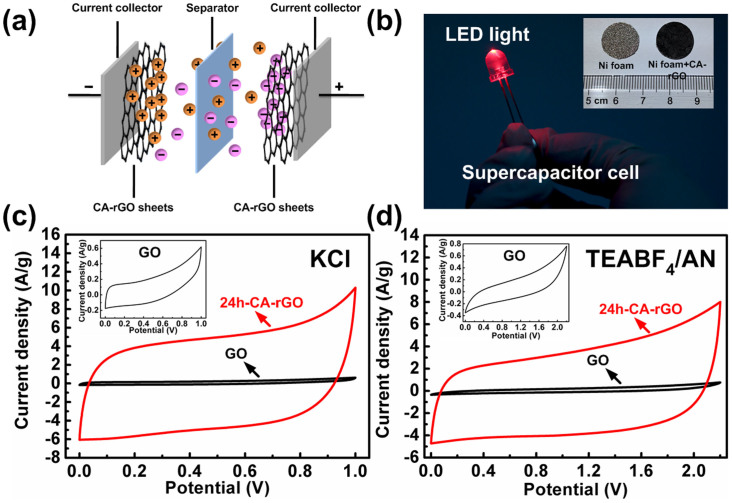
Supercapacitor application of CA-rGO. (a) Schematic diagram of the rGO-based double-layer supercapacitor. (b) Digital photographs of an LED light powered by a supercapacitor cell. Inset: digital photograph of a bare Ni foam before and after being coated with 24h-CA-rGO sheets. CV curves of the supercapacitors using GO and 24h-CA-rGO working electrodes in (c) 1.0 M KCl and (d) 1.0 M TEABF_4_/AN electrolytes tested at a scan rate of 100 mV/s.

Graphene-based structures have been recognized as quite promising active materials for supercapacitors (*i.e.*, EDLCs) due to graphene's huge specific surface area and high electrical conductivity^47^,^48^. Figs. 5a and b show a schematic of the graphene-based EDLCs and the digital picture of an LED light powered by a supercapacitor cell. EDLCs store charges electrostatically via reversible ion adsorption at the electrode/electrolyte interface, where the Ohmic resistance of the active materials will obviously influence the charge transport during the charge/discharge processes. To this respect, the CA-rGO sheet with an obviously improved conductivity than that of the parent GO, due to the restoration of π–π conjugated structure in graphene sheets, is therefore expected to show attractive EDLC properties.

Figs. 5c and d show the cyclic voltammetry (CV) curves of GO and 24h-CA-rGO based working electrodes employing KCl and tetraethylammonium tetrafluoroborate in acetonitrile solvent (TEABF_4_/AN) as the aqueous and organic electrolytes, respectively. The CV curves of 24h-CA-rGO (scan rate: 100 mV/s) show obviously larger CV areas, and correspondingly, higher specific capacitances (KCl: 96 F/g; TEABF_4_/AN: 74 F/g) than those of the GO (KCl: 3.7 F/g; TEABF_4_/AN: 6.0 F/g). Compared with the quite distorted shape of the CV curves of GO, those of 24h-CA-rGO were much closer to the quasirectangular shape, indicating the faster charging and discharging responses to the applied potential due to the significantly improved material conductivity after CA reduction. According to the Nyquist plots obtained from the electrochemical impedance spectroscopy (EIS) tests (Supplemental Information, Fig. S5), the imaginary component presents a sharp increase with a near-vertical line at low frequencies, confirming the predominant EDLCs.

The charge/discharge curves of the capacitors obtained at different current densities (1, 5, and 10 A/g) can be found in the Supplementary Information (Fig. S6). At a current density of 1 A/g, the specific capacitances of 24h-CA-rGO were calculated as 136 and 92 F/g for aqueous and organic electrolytes, respectively. The specific capacitance of 24h-CA-rGO was comparable to the ones using rGO prepared by toxic HH and significantly higher than those using non-toxic alcohol-reduced rGOs^22^,^49^. This observation could be somehow related to the difference in the specific surface areas of various active materials. The Brunauer–Emmett–Teller (BET) specific surface area of 24h-CA-rGO was measured as 122 m^2^/g (see N_2_ adsorption/desorption analysis in the Supplemental Information, Fig. S4), which is higher than that of the rGO reduced by alcohols (5.8–35.9 m^2^/g)^22^ while lower than that of rGO reduced by HH (normally 400–700 m^2^/g)^12^,^50^. It could be attributed to the easy formation of restacked rGO nanosheets during the reduction process with a relatively long reduction time due to the poorer reducing capabilities of alcohols and CA than that of HH^22^. The CV curves of 24h-CA-rGO supercapacitors at different scan rates (10, 20, 50, 100, and 200 mV/s) are presented in the Supplementary Information (Fig. S7). With an increasing scan rate from 20 to 200 mV/s, the capacitance retention of 24h-CA-rGO in aqueous electrolyte was 68% (from 126 to 86 F/g), close to that of HH (78%)^49^, indicating a good rate performance. The above results demonstrate the good electrochemical properties of CA-rGO and its high potential for energy storage applications. Further improvements on the capacitive behavior of CA-rGO appear likely through the optimization of material preparation and supercapacitor assembly.

In summary, rGO was successfully prepared with CA as the reducing agent. The rGO has been proved to have high a C/O ratio and a low oxygen content. The demonstrated electronic gas sensors with rGO as the sensing materials show fast and large responses to different gases under room temperature. The rGO has also been used as the active materials in EDLCs and the capacitors show comparable specific capacitance with that of hydrazine-reduced GO. We believe the rGO prepared with CA could be used in many environmental and energy applications, *e.g.*, sensors, supercapacitors, batteries, and catalysis. And this green reduction method could be attractive for facile large-scale manufacturing of graphene materials with a low cost.

## Methods

### Synthesis of GO

GO was synthesized following a modified Hummer's method^51^. In a typical procedure, 1 g natural graphite powder (XFNANO Materials Tech) was dispersed in 25 mL concentrated sulfuric acid at room temperature and the mixture was cooled down to 0°C in an ice bath. Subsequently, 3.5 g potassium permanganate (Sinopharm Chemical Reagent) was slowly added, and a 2 h stirring was conducted in a 35°C water bath followed by adding 100 mL deionized water. Then, 8 mL hydrogen peroxide solution (30 wt.% aqueous solution) was added until the color of the reaction mixture turned to bright yellow. Dilute hydrochloric acid solution (10% by volume) and deionized water were used to wash and remove the excess manganese salt and acids in the product. The product powder was obtained from centrifugation (8,000 r.m.p., 10 min) after repeating the washing process for four times. Finally, the product GO powder was dried at 35°C under vacuum.

### Reduction of GO with CA

The as-dried GO powder (100 mg) was dispersed in 1000 mL deionized water, followed by an ultrasonication for 1.5 h (FB15150, 300w, Fisher, Scientific). Different amounts of CA powder (Huilin Bio Tech) was added into the GO aqueous solution (concentration: 0.1 mg/mL) at room temperature. The pH of the mixture was measured after 10 minutes of mixing. The mixture was then heated to 95°C in an oil bath with the assistance of magnetic stirring for reaction. The corresponding products with different reduction time of 2, 12, and 24 hours were labeled as 2h-CA-rGO, 12h-CA-rGO, and 24h-CA-rGO, respectively. The resulting suspension was collected by vacuum filtration and washed with deionized water and ethanol for 10 times. Finally, rGO was collected after drying under vacuum condition. For comparison, reduction of GO with HH was also conducted. Briefly, 100 mL of GO dispersion (concentration: 1 mg/mL) was mixed with 1 mL hydrazine hydrate (98% from Sigma Aldrich), which was then kept in a 95°C oil bath and stirred for 24 hours. The as-obtained sample was labeled as HH-rGO.

### Material characterizations

TEM images and selected area electron diffraction (SAED) were obtained with a Technai G2 F30 S-Twin TEM (Philips-FEI). A Hitachi S-4800 SEM was used for SEM characterization at an acceleration voltage of 10 kV. UV-vis spectra were recorded on a Shimadzu UV-2550 spectrophotometer (Kyoutofu, Japan). FTIR spectra were carried out on a Nicolet 5700 FTIR spectrometer. XRD patterns were recorded with a XRD-6000 Diffractometer using Cu Kα Radiation (λ = 0.15425 nm, Shimadzu). XPS measurement was performed on a VG Escalab Mark II system employing a monochromatic Mg Kα X-Ray source (hm = 1,253.6 eV, West Sussex). The Raman spectra were taken with a DXR 532 Raman spectrometer (Thermo Fisher Scientific) in an excitation wavelength of 532 nm at room temperature. AFM images were taken on a MultiMode AutoProbe CP/MT Scanning Probe Microscope (Veeco Instruments, Woodbury, NY) operating in the tapping mode. TGA was performed using a Thermogravimetric Analyzer (Perkin Elmer, USA) under argon atmosphere with a flow rate of 100 mL/min. N_2_ adsorption-desorption measurements were carried out at 77.4 K using a Quantachrome Autosorb gas-sorption system (AUTOSORB-IQ-MP). Electrical conductivity measurement was carried out on a HALL5500 digital four-point probe system (Bio-Rad Co., USA). The rGO films for electrical conductivity measurement were prepared by filtration of rGO suspensions prepared in Dimethylformamide (DMF). The contact angles of the samples were measured by using a DropMeter™ Professional A-200 digital goniometer.

### Gas sensor tests

The details of the gas sensor fabrication were reported in our previous studies^44^,^46^. In a typical sensor, 0.5 μL 24h-CA-rGO suspension (0.1 mg/mL) was pipetted on the sensor electrode and dried under room temperature in air. To study the conductivity and FET characteristics of the rGO sensor, direct current and FET measurements were carried out using a Keithley 2602 source meter. The direct current measurement was performed by recording the drain current when ramping the drain-source voltage *V_d_* from −1.0 to +1.0 V (with a step of 0.1 V); while the FET measurement was performed by recording the drain current when ramping the gate voltage *V_g_* from −40 to +40 V (with a step of 0.1 V). The gas sensing performance of as-fabricated rGO was characterized against low-concentration NO_2_ (100 ppm) and NH_3_ (1%) diluted in dry air. Variations in the electrical conductance of rGO were monitored by simultaneously applying a constant dc voltage and recording the change in current passing through rGO sheets, which were exposed periodically to clean air, target gas, and clean air (flow rate: 2 lpm for all gases).

### Supercapacitor tests

Supercapacitors with GO and rGO as the active materials were assembled into a two-electrode system with nickel foam as the current collector. The test coin cell consisted of a metal cap, a metal case with polymer seal, a spring, two stainless steel spacers, two current collectors coated with active materials, and a membrane separator. 1.0 M KCl (Sigma Aldrich) and 1.0 M TEABF_4_/AN (Sigma Aldrich) were used as the aqueous and organic electrolytes, respectively. For supercapacitors with organic electrolyte, coin cells were assembled in the vacuum glove box with argon atmosphere to avoid oxygen and moisture^52^. To prepare film-like rGO-based active materials, the rGO suspension was treated by a 1-h vacuum filtration through a membrane filter of 0.22 μm in pore size, followed by a 12-h freeze-drying process which could benefit the formation of an rGO film with a relatively large size. The GO film was fabricated by 12-h vacuum filtration of the GO dispersion through the same membrane filter. No binder or conductive agent was applied, and the rGO and GO films peeled off the membrane were used for supercapacitor test cell assembly. The mass of the rGO and GO films in a single electrode was identical (~1 mg). The capacitive behavior of supercapacitors was tested by CV, galvanostatic charge/discharge, and EIS on an electrochemical workstation (PGSTAT302N, Metrohm Autolab B.V.) at room temperature. Based on the CV curves, the specific capacitance of a single electrode (*C*_cv_, unit: F/g) was calculated as: 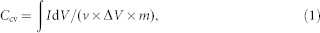
where *I* is the response current (unit: A), *v* is the potential scan rate (unit: V/s), *V* is the potential window (unit: V), and *m* is the mass of active materials on the single electrode, respectively. With the galvanostatic charge/discharge plots, the specific capacitance of a single electrode (*C*_g_, unit: F/g) was calculated as: 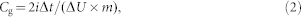
where *i* is the constant discharge current (unit: A), *t* is the discharge time (unit: s), and *U* is the voltage drop upon discharging (unit: V).

## Author Contributions

Z.B., S.M. and K.C. designed this research; X.S. synthesized the GO and rGO materials; S.M. conducted the gas sensing tests; H.Y. and J.Q. carried out the supercapacitor tests; X.S. and J.Y. analyzed the data; Z.B., S.M. and J.C. drafted the manuscript; and all authors commented on the final manuscript.

## Supplementary Material

Supplementary Information Green Preparation of Reduced Graphene Oxide for Sensing and Energy Storage Applications
