# Supplementary material for: Green preparation of reduced graphene oxide for sensing and energy storage applications
Source: Sci Rep. 2014 Apr 15;4:4684. doi: 10.1038/srep04684 (PMC3986759; doi:10.1038/srep04684)
Supplement: Supplementary Information — Green Preparation of Reduced Graphene Oxide for Sensing and Energy Storage Applications [file srep04684-s1.pdf]

Supplementary Information

Green Preparation of Reduced Graphene Oxide for Sensing and Energy Storage Applications

Zheng Bo[1], Xiaorui Shuai[1], Shun Mao[2]*, Huachao Yang[1], Jiajing Qian[1], Junhong Chen[2]*, Jianhua Yan[1] & Kefa Cen[1]

[1]State Key Laboratory of Clean Energy Utilization, Institute for Thermal Power Engineering, Department of Energy Engineering, Zhejiang University, Hangzhou, Zhejiang Province, 310027, China

[2]Department of Mechanical Engineering, University of Wisconsin-Milwaukee, 3200 North Cramer Street, Milwaukee, WI 53211, USA

Contents

1. AFM data of 24h-CA-rGOs.

2. FTIR and UV-vis absorption spectra of GO and rGOs.

3. TGA analysis of GO, 2h-CA-rGO, 12h-CA-rGO, and 24h-CA-rGO.

4. BET surface area study of 24h-CA-rGO.

5. Supercapacitor tests of 24h-CA-rGO.

6. Comparison of rGO reduced by different reducing agents.

1. AFM data of 24h-CA-rGOs.

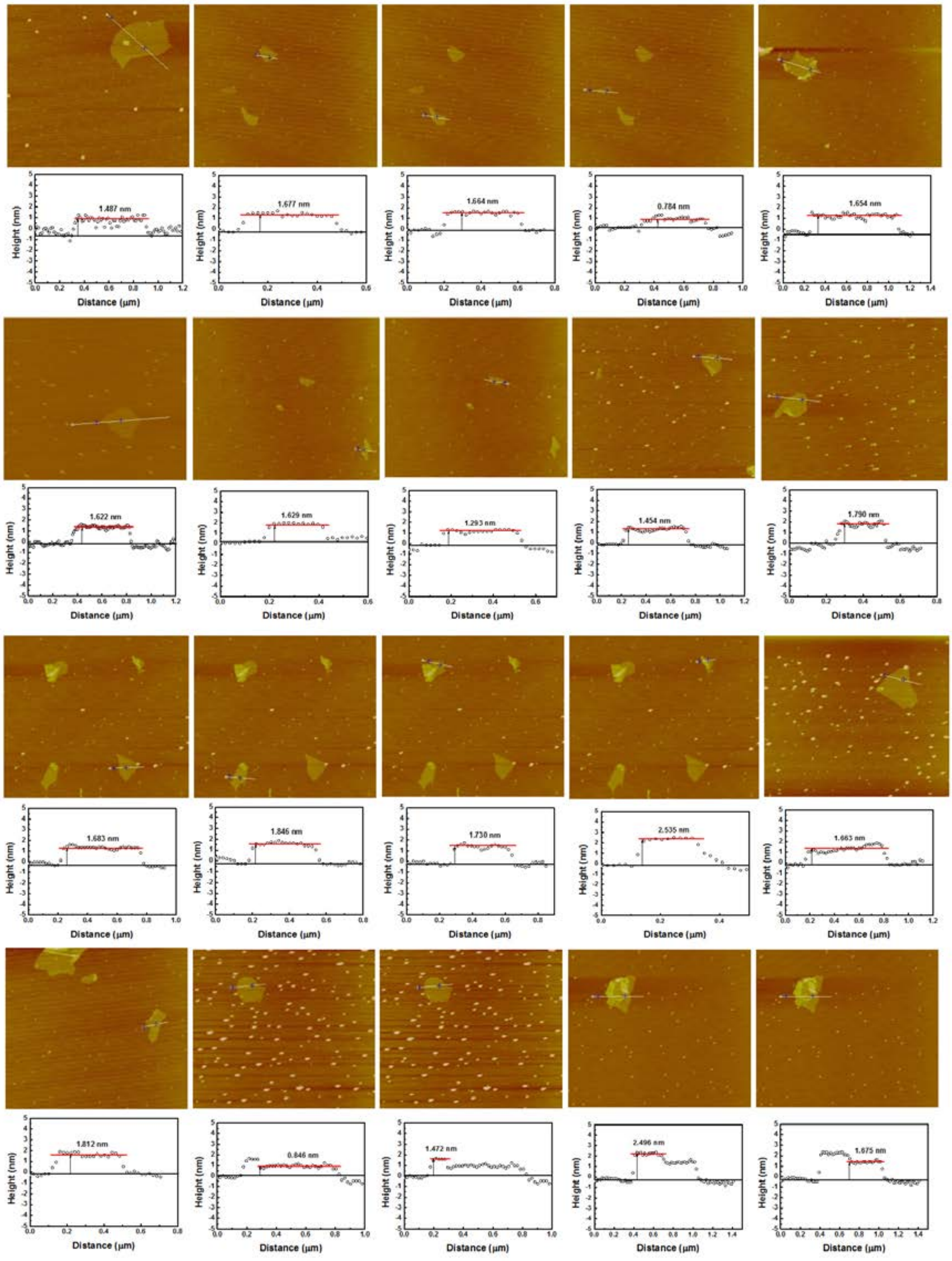

Figure S1. AMF images and corresponding height profiles of randomly selected 24h-CA-rGOs.

2. FTIR and UV-vis absorption spectra of GO and rGOs.

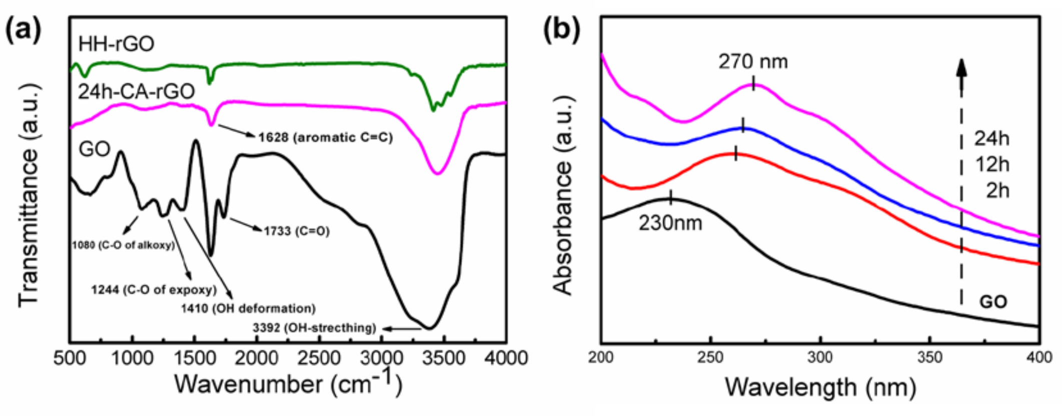

Figure S2. (a) FTIR spectra of GO, 24h-CA-rGO, and HH-rGO, respectively. (b) UV-vis absorption spectra of GO aqueous dispersion as a function of reduction time.

Fig. S2 shows the FTIR spectra of GO, 24h-CA-rGO, and HH-rGO. The FTIR spectrum of GO (Fig. S2a) shows a OH stretching mode at 3,382 cm^{-1}, a C=O vibrational mode at 1,733 cm^{-1}, a deformation peak of OH groups at 1,410 cm^{-1}, and stretching vibration bands C-O (epoxy and alkoxy) at 1,244 and 1,080 cm^{-1}, respectively, indicating the existence of abundant oxygen functional groups at the edges and the basal plane of GO. For the 24h-CA-rGO, the adsorption intensities of oxygen functional groups decreased dramatically and the peaks from epoxy and alkoxy almost disappeared. The above results suggest the successful removal of oxygenated groups and restoration of the carbon plane in 24h-CA-rGO. As a reference, the FTIR spectrum of 24h-CA-rGO was found to be quite similar to that of HH-rGO.

The reduction process as a function of time was monitored by UV-vis absorption spectroscopy. Fig. S2b shows the UV-vis absorption spectra of GO, 2h-CA-rGO, 12h-CA-rGO, and 24h-CA-rGO suspensions. The UV-vis absorption spectrum of GO presented a characteristic peak at 230 nm, originated from the π-π* transitions of carbon−carbon (C=C) bonds. The position of this absorption peak gradually red shifted to a higher wavelength with the increase in the reduction time, indicating the successful restoration of highly conjugated electronic structure. Knowing that the maximum absorption peaks of CA locate at 312 nm and 286 nm, it is reasonable to

conclude that the above observation is attributed to the GO reduction rather than the interference of the reducing agent. Compared with other rGO counterparts using green reducing agents, such as baker's yeast (264 nm), Escherichia coli (267 nm), and glycine (267 nm), the UV-vis absorption spectrum of 24h-CA-rGO presented a higher wavelength for the carbon−carbon bonds, indicating a higher reduction efficiency of CA.

3. TGA analysis of GO, 2h-CA-rGO, 12h-CA-rGO, and 24h-CA-rGO.

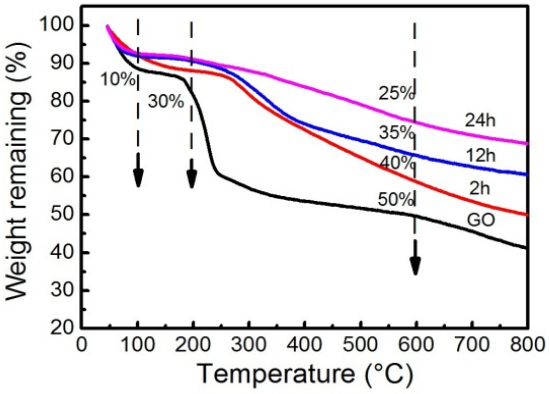

Figure S3. Normalized TGA curves of GO and CA-rGOs for different reduction time.

The thermal stabilities of GO, 2h-CA-rGO, 12h-CA-rGO, and 24h-CA-rGO were evaluated by TGA. Fig. S3 shows the TGA curves of the above samples from 45 to 800 °C with a heating rate of 10 °C/min. For GO, the initial mass loss of ~10% at around 100 °C was due to the loss of adsorbed water. The subsequent mass loss of ~30% (200 °C) was attributed to the pyrolysis of the labile oxygen-containing functional groups. For the temperature range above 300 °C, a slow mass loss was observed, probably owing to the decomposition of more stable oxygen functional groups. Compared with GO, the 24h-CA-rGO sample shows no significant mass loss at 200 °C, indicating the successful removal of oxygen-containing groups during the reduction. And the mass loss of the 24h-CA-rGO (~25%) was much lower than that of GO (~50%) at 600 °C. This TGA results further present evidence for the successful reduction of GO using CA as the reducing agent.

4. BET specific surface area of 24h-CA-rGO.

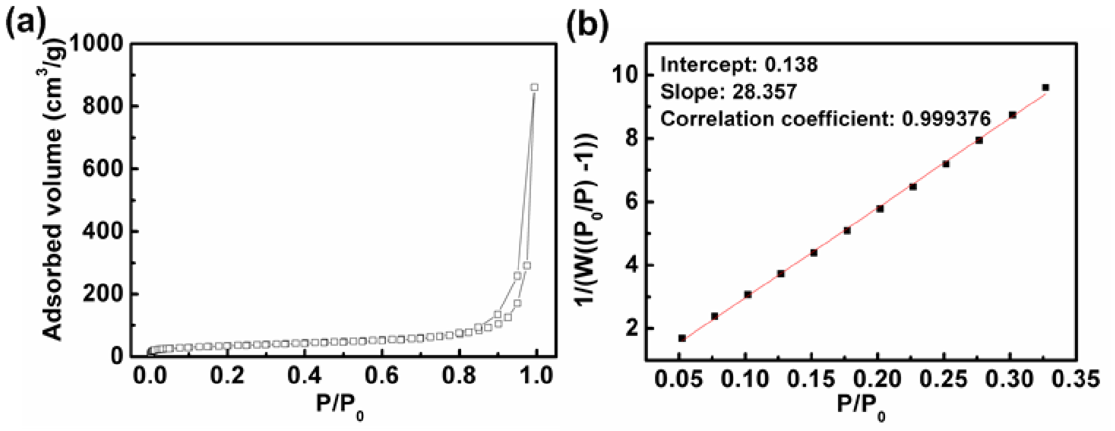

Figure S4. (a) N_2 isotherm curves at 77.4 K; and (b) BET surface area plot (P/P_0 range: 0.05-0.35) of 24h-CA-rGO. W is the quantity of adsorbed gas; P and P_0 are the equilibrium and the saturation pressure of the adsorbate. The BET specific surface area of 24h-CA-rGO was measured as 122 m^2/g.

5. Supercapacitor tests of 24h-CA-rGO.

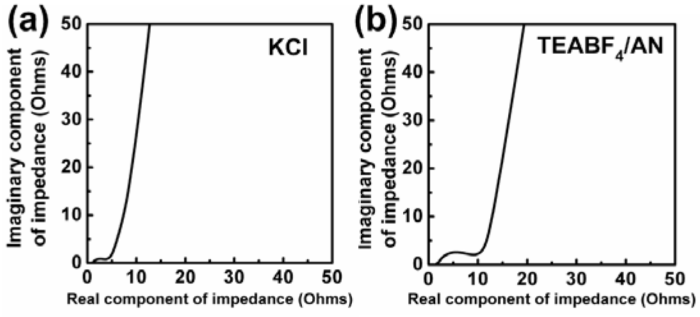

Figure S5. Nyquist plots of 24h-CA-rGO supercapacitors tested in (a) KCl and (b) $TEABF_4$/AN electrolytes.

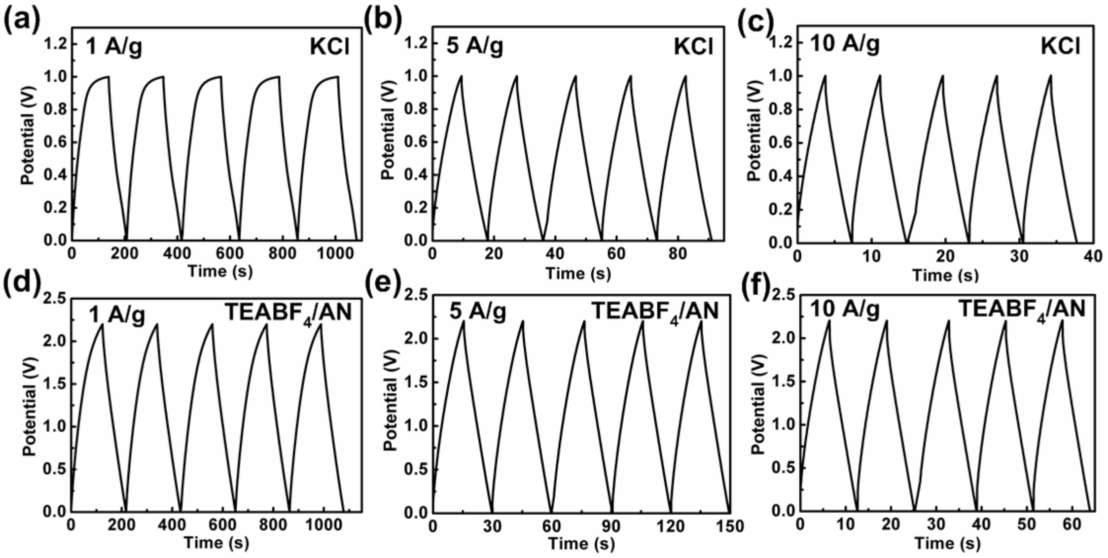

Figure S6. Galvanostatic charge/discharge plots of 24h-CA-rGO supercapacitors using aqueous and organic electrolytes at current densities of 1, 5, and 10 A/g.

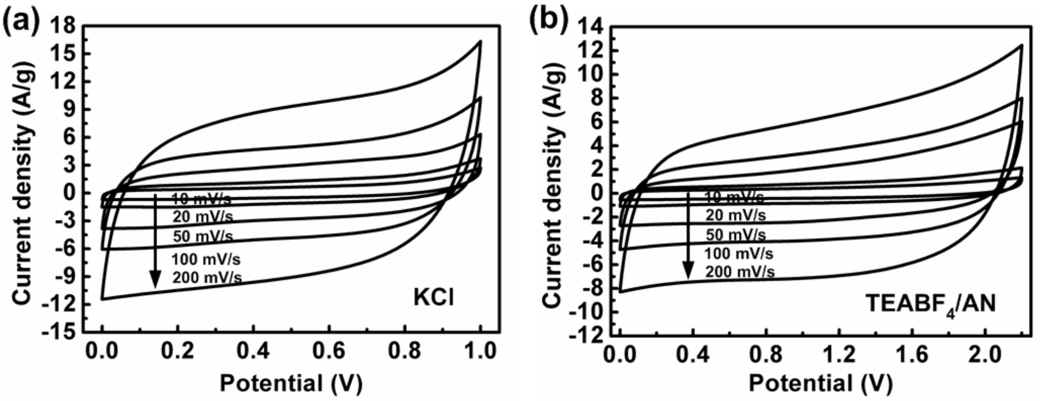

Figure S7. CVs of 24h-CA-rGO supercapacitors using aqueous and organic electrolytes at different scan rates between 10 and 200 mV/s.

6. Comparison of rGO reduced by different reducing agents.

Table S1 Characterizations of rGO prepared by various green reducing agents.

Reducing agents	C/O ratio	Category	UV-vis peak wavelength (nm)	I_D/I_G	Ref.
Tannin acid	2.44	Phenols	274	1.15	S1
Tea polyphenol	3.10	Phenols	271	/	S2
Gallic acid	3.89-5.28	Phenols	270-273	1.86-1.92	S3
Methanol	4.00	Alcohols	/	1.15	S4
Natural cellulose	5.47	Polysaccharides	269	1.53	S5
Sodium-citrate	5.60	Organic acid salts	265	/	S6
L-Ascorbic acid	5.70	Organic acids	264	>1	S7
Baker's yeast	5.90	Microbes	263	1.44	S8
Ethanol	6.00	Alcohols	/	1.10	S4
Isopropanol	6.90	Alcohols	/	1.25	S4
Zinc	6.02-7.39	Metals	265-265	1.42-1.51	S9
**CA**	**7.15**	**Phenols**	**270**	**1.15**	**This study**
Iron	7.90	Metals	300	0.32	S10
Glycine	11.14	Amino acids	267	1.09	S11
Vitamin C	12.50	Organic acids	268	/	S12
Aluminum	18.61	Metals	/	1.81	S13
Benzyl alcohol	30.00	Alcohols	/	1.20	S4

References

S1. Lei, Y. D., Tang, Z. H., Liao, R. J. & Guo, B. C. Hydrolysable tannin as environmentally friendly reducer and stabilizer for graphene oxide. Green Chem. 13, 1655-1658 (2011).

S2. Wang, Y., Shi, Z. X. & Yin, J. Facile Synthesis of Soluble Graphene via a Green Reduction of Graphene Oxide in Tea Solution and Its Biocomposites. ACS Appl. Mater. Interfaces 3, 1127-1133 (2011).

S3. Li, J., Xiao, G. Y., Chen, C. B., Li, R. & Yan, D. Y. Superior dispersions of reduced graphene oxide synthesized by using gallic acid as a reductant and stabilizer. J. Mater. Chem. A 1, 1481-1487 (2013).

S4. Dreyer, D. R., Murali, S., Zhu, Y. W., Ruoff, R. S. & Bielawski, C. W. Reduction of graphite oxide using alcohols. J. Mater. Chem. 21, 3443-3447 (2011).

S5. Peng, H. D., Meng, L. J., Niu, L. Y. & Lu, Q. H. Simultaneous Reduction and Surface Functionalization of Graphene Oxide by Natural Cellulose with the Assistance of the Ionic Liquid. J. Phys. Chem. C 116, 16294-16299 (2012).

S6. Wan, W. B. et al. "Green" reduction of graphene oxide to graphene by sodium citrate. New Carbon Mater. 26, 16-20 (2011).

S7. Zhang, J. L. et al. Reduction of graphene oxide via L-ascorbic acid. Chem. Commun. 46, 1112-1114 (2010).

S8. Khanra, P. et al. Simultaneous bio-functionalization and reduction of graphene oxide by baker's yeast. Chem. Eng. J. 183, 526-533 (2012).

S9. Yang, S. et al. A facile green strategy for rapid reduction of graphene oxide by metallic zinc. RSC Adv. 2, 8827-8832 (2012).

S10. Fan, Z. J. et al. Facile Synthesis of Graphene Nanosheets via Fe Reduction of Exfoliated Graphite Oxide. ACS Nano 5, 191-198 (2011).

S11. Bose, S., Kuila, T., Mishra, A. K., Kim, N. H. & Lee, J. H. Dual role of glycine as a chemical functionalizer and a reducing agent in the preparation of graphene: an environmentally friendly method. J. Mater. Chem. 22, 9696-9703 (2012).

S12. Fernandez-Merino, M. J. et al. Vitamin C Is an Ideal Substitute for Hydrazine in the Reduction of Graphene Oxide Suspensions. J. Phys. Chem. C 114, 6426-6432 (2010).

S13. Fan, Z. J. et al. An environmentally friendly and efficient route for the reduction of graphene oxide by aluminum powder. Carbon 48, 1686-1689 (2010).
